# Atrial Fibrillation as an Independent Predictor of Myocardial Ischemia

**DOI:** 10.3390/medicina61020337

**Published:** 2025-02-14

**Authors:** Aris Bechlioulis, Aidonis Rammos, Athanassios Papadopoulos, Paraskeni Zotou, Sotiria Alexiou, Areti Kekiopoulou, Lampros K. Michalis, Katerina K. Naka, Chrissa Sioka, Christos Katsouras

**Affiliations:** 1 2nd Department of Cardiology, University Hospital of Ioannina, 455 00 Ioannina, Greece; 2 Department of Medical Physics, University Hospital of Ioannina, 455 00 Ioannina, Greece; 3 Department of Nuclear Medicine, University Hospital of Ioannina, 455 00 Ioannina, Greece

**Keywords:** atrial fibrillation, coronary artery disease, myocardial perfusion imaging, myocardial ischemia, single-photon emission computed tomography

## Abstract

*Background and Objectives:* Atrial fibrillation (AF) and coronary artery disease (CAD) are highly prevalent cardiovascular conditions. This study investigated the role of AF in myocardial ischemia, as assessed with myocardial perfusion imaging (MPI), in patients with suspected stable CAD. *Materials and Methods:* Our retrospective study included 259 individuals with a negative medical history of CAD who underwent 99mTc tetrofosmin MPI—single-photon emission computed tomography (SPECT)—for nonspecific symptoms to rule out stable CAD. *Results:* Of the enrolled patients, 90 MPIs were from patients with AF and 169 MPIs were from patients without AF. Semi-quantitative assessments of the extent and severity of perfusion abnormalities according to the summed stress score (SSS) and summed difference score (SDS) were conducted. It was found that patients with a history of AF, compared to patients without AF, were older (*p* < 0.001), of the male gender (*p* < 0.001), and had dyslipidemia (*p* = 0.019). History of AF was associated with increased SSS ≥ 4 (OR 5.12, *p* < 0.001) and SDS ≥ 2 (OR 2.66, *p* < 0.001). After adjustment for other risk factors, AF remained an independent predictor of myocardial ischemia on MPI-SPECT. *Conclusions:* In the current study, an association of AF with extensive perfusion defects in MPI-SPECT studies was found in patients with clinically suspected CAD independently of common cardiovascular risk factors.

## 1. Introduction

Coronary artery disease (CAD) is a leading cause of morbidity and mortality worldwide, with variable phenotypes ranging from acute coronary syndromes to stable effort angina that may be associated with impaired quality of life in case of persistent anginal symptoms and the subsequent need for frequent hospitalizations [1]. Individuals with established ischemic heart disease may appear asymptomatic for long periods of time, but occasionally, periods of myocardial ischemia symptoms exacerbation occur either at rest or during periods of minimal or heavy physical activity, due to coronary atherosclerosis progression and/or increased myocardial blood demand. These syndromes may manifest with minimal or nonspecific symptomatology and can be associated with the absence of significant epicardial coronary artery stenosis due to either microvascular dysfunction or abnormal coronary arterial vasomotor activity [2].

The presence of typical or atypical angina symptoms often requires evaluation that frequently involves the performance of myocardial stress tests depending on the per-case pre-test probability of the presence of CAD [3]. Cardiac stress myocardial perfusion imaging with single-photon emission computed tomography (MPI-SPECT) can assess coronary perfusion, myocardium viability, and exercise capacity [4]. Stress is induced either pharmacologically or by physical exercise. The modern MPI test provides an accurate estimation of myocardial perfusion defects and may guide decisions regarding the need for invasive coronary angiography after consideration of the patient’s clinical syndrome [4]. Furthermore, it offers valuable information for risk stratification, prognosis, and treatment options that, on many occasions, may be independent of the presence of significant epicardial CAD. Based on these characteristics, MPI-SPECT has been supported by current practice guidelines in everyday clinical practice [5]. MPI-SPECT has been used as a diagnostic and prognostic tool for patients presenting with symptoms of suspected CAD [6], with good diagnostic accuracy (area under the curve [AUC] 0.74; sensitivity 74%, and specificity 73%) [7]. It has been reported that compared with stress echocardiography, MPI-SPECT has better sensitivity and specificity; however, it may be inferior to other less widely available and costly modalities such as stress cardiac magnetic resonance and positron emission tomography [8,9].

Cardiac arrhythmias are a frequent finding in patients suffering from heart diseases, including CAD, and are found in the majority of patients with CAD during the course of the disease, either in the acute ischemic phase or the chronic stable phase [10]. Atrial fibrillation (AF) is the most common arrhythmia in the general population, and its incidence and prevalence continue to increase in modern times [11]. In a recent large association study, the presence of CAD was strongly associated with approximately 20% higher risk of AF incidence [12]. Ischemic heart disease alters the structure and function of the atrial myocardium, either through increased mechanical loading due to impaired ventricular function or through a direct effect of the ischemia on the electrophysiological properties of the cardiomyocytes, involving ischemic necrosis and replacement by fibrous tissue, and increased susceptibility to arrhythmias [13]. On the other hand, AF per se may increase oxygen consumption of the myocardium, creating a mismatch between supply and demand in patients with existing CAD. It may also promote the development of a so-called pro-atherogenic environment including increased inflammation and oxidative stress, activation of the renin–angiotensin–aldosterone and sympathetic systems, leading to early endothelial dysfunction and a prothrombotic state [13]. The epidemiological association and co-existence of AF and CAD in the same patients is undisputable. They share many common risk factors such as hypertension, obesity, and type 2 diabetes mellitus and they both increase with age [14,15]. Thus, it has been argued that AF and CAD can aggravate each other in a vicious cycle [16].

The role and practical application of the MPI-SPECT stress test for CAD screening in patients with AF has not been well demonstrated, although there has been no implication for current practice guidelines. A retrospective study reported similar prevalence of perfusion defects and a similar role in the clinical prognosis of patients with suspected CAD irrespective of the presence of AF history after adjustment for various confounders [17]. Nevertheless, the presence of AF history in symptomatic patients with suspected CAD or a prior history of CAD who underwent MPI-SPECT was significantly associated with cardiac death across all categories of MPI-SPECT results, even in multivariable analysis (*p* = 0.001) [18]. On the other hand, the presence of myocardial perfusion defects in MPI-SPECT studies in patients with AF compared to age- and gender-matched controls was less often related to significant epicardial CAD in subsequent coronary angiography, leading to confusion regarding the interpretation of MPI-SPECT results in the presence of AF [19].

The aim of the current study was to investigate the association of persistent/permanent AF with the extent of perfusion defects in MPI-SPECT study in a cohort of patients evaluated for suspected stable CAD.

## 2. Methods

### 2.1. Study Population

This single-center study included adult patients who underwent a myocardial perfusion imaging test using single-photon emission computed tomography for suspected stable coronary artery disease. Two hundred and fifty-nine individuals who underwent MPI-SPECT in the Nuclear Medicine Department of the University Hospital of Ioannina were included in the current study analysis. Among them, 169 patients had no history of AF and comprised the control group (CG). The remaining 90 patients had persistent or permanent AF during the MPI study and were defined as the study group. Exclusion criteria for all individuals included a history of previously diagnosed CAD, the presence of heart failure with reduced or preserved left ventricular ejection fraction, asymptomatic individuals with left ventricular ejection fraction <40%, the presence of profound bradycardia (<40 beats per min), high degree of AV block, myocardial conduction abnormalities (left or right bundle branch block), end-stage renal disease, active cancer, chronic medical conditions, or any acute symptomatic medical illness, systolic arterial blood pressure at rest >200 mmHg or <90 mmHg or diastolic blood pressure >110 mmHg, and history of a previous MPI-SPECT study. Known CAD was considered if any of the following was present: history of myocardial infarction, percutaneous coronary intervention, coronary artery bypass grafting, invasive or noninvasive (computed tomography) coronary angiography with at least one luminal diameter stenosis >50% in any epicardial coronary artery with a diameter ≥2 mm or stenosis ≤50% associated with invasive physiological assessment with pressure wire techniques and abnormal results. Patients were considered to have end-stage renal disease if they had advanced chronic kidney disease (stage 4 or 5) or they were on dialysis. Active cancer was defined as any cancer under treatment or recent (within the previous 6 months) diagnosis, any metastatic cancer, and any cancer left without therapy.

The Hospital’s Ethics Committee approved the protocol of this study. All studies were performed based on clear medical indications. All participants provided their written informed consent to participate in this study. All data used for analysis, medical information, and results from MPI-SPECTs were preserved and analyzed anonymously.

### 2.2. Study Protocol

MPI-SPECT study was performed according to the guidelines of the European Association of Nuclear Medicine (EANM) [20]. Medical history information was obtained from all patients following a short interview before the study; classic cardiovascular risk factors and persistent or permanent AF were recorded. The heart rate was not consistently recorded in all patients and hence this parameter could not be included in the association analysis. Before testing, any products containing methylxanthines should have been avoided for at least 12 h and nothing should have been eaten during the last 3 h.

A single-day protocol was used, with stress and rest images acquired in that order, approximately two hours apart [21]. Specifically, each participating examinee was intravenously infused with the radiopharmaceutical 99mTc-Tetrofosmine, during both the first phase (stress) and the second phase (rest). The uptake of the perfusion tracer was related to the perfusion of the myocardial tissue. Although the perfusion imaging might be performed utilizing thallium-201 or technetium-99 m labeled radiopharmaceuticals, the present study introduced Tc-99m tetrofosmin as a tracer, an MPI radiopharmaceutical labeled in-house according to the manufacturer’s instructions.

Before the study’s initiation, blood pressure and oxygen saturation were measured in all patients and a 12-lead electrocardiogram was acquired. A stress test was performed with an intravenous dipyridamole infusion (0.56 mg/kg body weight over 4 min). The subject was intravenously infused for the stress phase with 8 mCi Tc-99m tetrofosmin and with 20 mCi Tc-99m tetrofosmin for the rest phase. Subsequently, after the lapse of 20 min, the patient was positioned on the SPECT γ-camera for imaging.

The camera consists of a modern dual-head General Electric (GE) OPTIMA 950 γ-camera with a viewing angle of 90°. A 180° acquisition was preferred for non-attenuation-corrected images because it provided better spatial resolution, higher contrast, and less attenuation. SPECT images were acquired using a high-resolution collimator. A step-and-shoot acquisition with 64 stops separated by 3° was used. The duration of the acquisition at each stop was set equal to 25 s/image.

The series projections were first reviewed for possible artifacts due to attenuation and patient motion, image processing issues, and overall image quality. Prior to reconstruction, the SPECT projection data were reviewed by experienced users as a cine display to detect patient motion. Significant patient motion during image acquisition may lead to the repentance of the acquisition process or the application of specific software for image repair. The reconstruction of the images was carried out using the Iterative Reconstruction algorithm, incorporating the widely used ordered subsets expectation maximization (OSEM).

**Figure 1:**
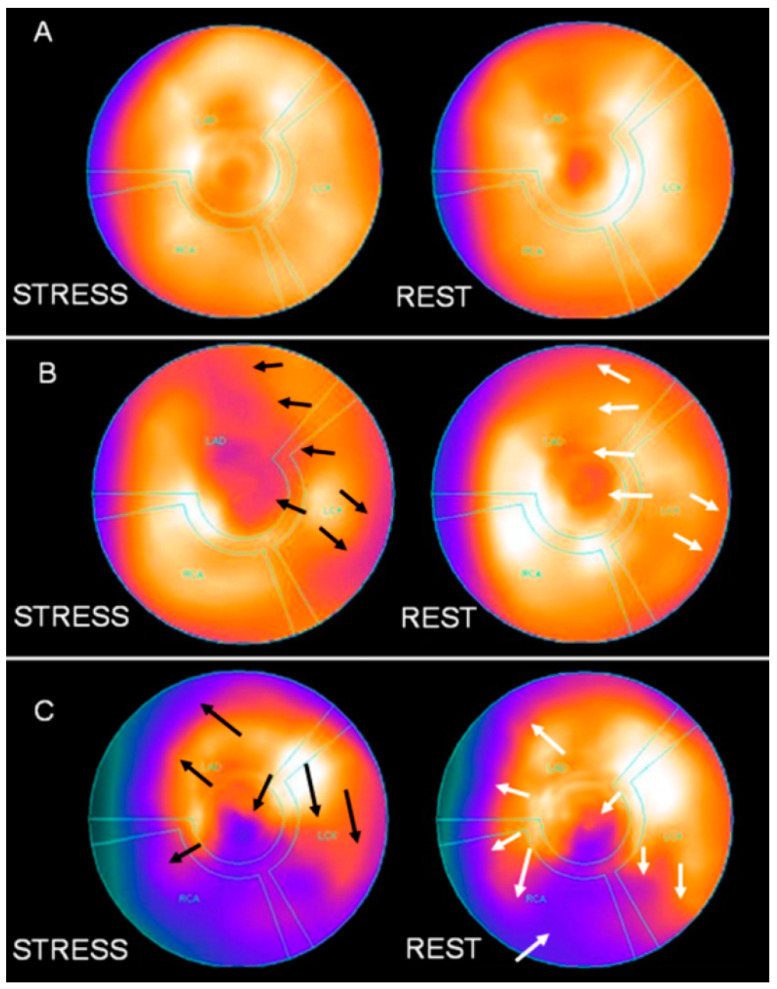
A summed stress score (SSS) > 3 indicates an abnormal MPI-SPECT, considered with myocardial ischemia or a myocardial infarct. Additionally, according to the score, myocardial ischemia may be graded as mild (4 ≤ SSS < 9), moderate (9 ≤ SSS < 14), or severe (SSS > 14)**.** Summed rest score (SRS) indicates defects at rest, defects attributed to a scar, or defects attributed to a hibernating myocardium. Finally, the difference between SSS and SRS indicates the reversibility of ischemia induced by stress (exercise or pharmacologic stress such as in our cases). Here, we present three different MPI-SPECTs. Black arrows show the defects in stress and white arrows indicate the reversibility (complete or partial) in MPI-SPECT. (**A**) The MPI-SPECT did not reveal any defects and it was diagnosed as normal. (**B**) The MPI-SPECT revealed mild ischemia involving the cardiac apex of the anterior and basal lateral cardiac wall. The SSS and SRS were 7 and 0, respectively. The SDS was 7, indicating a reversible myocardial ischemia. (**C**) The MPI-SPECT revealed severe ischemia, involving the cardiac apex, lateral apical, middle and basal infero-lateral, the inferior and the middle and basal infra-septal cardiac wall (SSS—23, SRS—10 and SDS—13), indicating a moderately reversible myocardial ischemia.

The recorded raw tomographic data pertaining to stress and rest were processed and reconstructed in the three planes (short axis, horizontal long axis, and vertical long axis) according to established algorithms and assessed by two experienced board-certified nuclear medicine physicians using a 17-segment polar map, as previously reported in both stress and rest images [22]. Each segment was scored on a scale of 0 to 4 depending on the degree of the perfusion deficit, with scores ranging from 0 to 64. A summed stress score (SSS) >3 was considered indicative of myocardial ischemia. The summed difference score (SDS = SSS − SRS) denoted the presence of reversible perfusion defects following pharmacologic stress. An SDS of 0–1 was considered to be within normal limits, 2–4 points consisted of mild ischemia, 5–6 points specified moderate ischemia, and 7 or more points indicated severe ischemia, i.e., significant stress perfusion deficit (Figure 1) [23].

Regarding the radiation exposure throughout the MPI-SPECT study, the applied protocol was optimized for the patient’s radiation exposure. By utilizing best practices, a significant reduction in radiation exposure was achieved. Such practices involved the usage of high-technology software and hardware, and the selection of multiple positions (e.g., both supine and prone) and weight-based dosing adjustments.

### 2.3. Statistical Analysis

Continuous parameters are presented as mean ± standard deviation or median (interquartile range). Dichotomous variables are presented as numbers (percent). Fisher’s chi-square test was used to compare dichotomous variables between the two groups. An unpaired *t*-test and a Mann–Whitney U test were used to compare continuous variables between the two groups. Logistic regression analysis was used for univariate and multivariate associations of AF with MPI-SPECT results (SSS and SDS). In multivariate analysis, the association of AF with SSS and SDS was adjusted for differences in studied demographic and clinical characteristics between the two groups. Spearman’s rho coefficient was used to assess the correlation of various studied parameters with increased SSS and SDS within each group of patients. *p* values were always two-sided, and a value of *p* < 0.05 was considered significant. The SPSS statistical software package (IBM SPSS Statistics, Version 23) was used.

## 3. Results

Two hundred fifty-nine consecutive patients who underwent an MPI-SPECT study for suspected CAD were finally enrolled. The majority of the total population were female patients (70%) with arterial hypertension (73%) and dyslipidemia (61%) while current smoking and diabetes were present in a minority of patients (28% and 26%, respectively). A high SSS > 3 and high SDS > 1 were found in 44% and 50%, respectively, of the entire population.

Among them, 169 patients had no history of AF and 90 patients had a history of AF. The descriptive characteristics of the two groups of patients with versus without a history of AF are shown in Table 1. Patients with a history of AF were older (79 ± 9 versus 61 ± 11 years, *p* < 0.001) and had a higher prevalence of male gender (49% versus 20%, *p* < 0.001) and dyslipidemia (71% versus 56%, *p* = 0.019). The proportion of patients with diabetes mellitus, smoking status and hypertension did not differ significantly in the two groups (*p* > 0.05 for all).

In logistic regression analysis, a history of AF was associated with higher SSS (i.e., SSS > 3) OR 5.12, *p* < 0.001, 95% CI (2.90, 9.04) and SDS (i.e., >1) OR 2.66, *p* < 0.001, 95% CI (1.54, 4.59); after adjustment for confounders, i.e., age, smoking, dyslipidemia, diabetes, hypertension, gender and weight, history of AF remained significantly associated with both SSS > 3 OR 4.60, *p* < 0.001, 95% CI (2.31, 9.18) and SDS > 1 OR 2.99, *p* = 0.001, 95% CI (1.53, 5.84).

In patients without history of AF, increased SSS > 3 was significantly associated with increased body weight r = 0.277, *p* = 0.001 and increased SDS > 1 was significantly associated with increased body weight r = 0.276, *p* = 0.001 and hypertension r = 0.131, *p* = 0.008. In patients with a history of AF, increased SSS > 3 was associated with male gender r = 0.359, *p* = 0.001. No parameter was significantly associated with increased SDS > 1 in patients with a history of AF.

## 4. Discussion

In the present study, it was demonstrated that AF was an independent predictor of severe perfusion defects, both at rest and after stress, in MPI-SPECT studies of patients with suspected CAD. Patients with AF had a higher prevalence of established atherosclerotic risk factors such as smoking, dyslipidemia, male gender, and increased age. However, the association of AF with impaired myocardial perfusion remained significant even after the adjustment for the above-mentioned risk factors. In the present study, AF was independently related to a 3–5-fold higher risk of extensive rest or stress-induced myocardial perfusion defects.

A high prevalence of CAD identified by invasive coronary angiography or computed tomography coronary angiography has been previously reported among non-valvular AF patients (>50%) [12,13,24] compared to the general population (appx 12–14%) and AF was shown to be an independent risk factor for chronic CAD and acute coronary syndromes [14,15]. In a most recent study involving AF patients preparing to undergo catheter ablation (mean age 60 years old appx), the prevalence of significant angiographic CAD in computed tomography coronary angiography was 20%, while critical CAD was evident in 7% [25]. A recent systematic review and meta-analysis demonstrated that AF was associated with a 1.54-fold increased risk of a future myocardial infarction associated with atherosclerotic CAD [16]. Furthermore, patients with diagnosed CAD and co-existing AF had more extensive angiographic CAD, as assessed by higher SYNTAX scores compared to CAD patients without AF [17]. On the other hand, in patients with established chronic CAD, the incidence of new onset AF was 5% in 5 years of follow-up (CLARIFY registry) and signified a worse clinical prognosis thereafter [26]. Morbidity and mortality were deemed to be significantly higher in patients with established CAD when it was associated with AF and this was accompanied by a higher risk of developing heart failure, although this relationship may be influenced by various confounding risk factors in CAD/AF patients, such as diabetes mellitus, hypertension, age, and obesity [14,26].

Based on the above data, a bidirectional etiological relationship between AF and CAD has been suggested and the notion that AF should be considered as a truly systemic vascular disease was previously emphasized [27]. Common risk factors that contribute to the development and progression of both AF and CAD are diabetes mellitus, hypertension, advancing age, dyslipidemia, obesity, smoking, and decreased physical activity [24] AF brings together several hemodynamic and systemic changes, including inflammation, oxidative stress, activation of the renin–angiotensin–aldosterone and sympathetic systems, as well as a prothrombotic state and endothelial dysfunction, which are also essential pathophysiological pathways in vascular atherosclerosis [13,27].

A high prevalence of CAD (>50%) identified by ICA or CTCA has been reported among non-valvular AF patients [28,29] compared to the general population, while AF was an independent risk factor for CAD and acute coronary syndromes (ACSs) [16,30]. An analysis of 4,371,714 individuals within 20 studies demonstrated that AF was linked to an increased risk of mortality in women rather than men (RR 1.12, 95% CI 1.07–1.17) and was associated with a statistically significant increase in the risk of cardiac events, heart failure, and stroke [31]. AF patients appear to have a higher prevalence of coronary artery disease compared to non-AF patients, and this has prompted some investigators to question if AF patients should always undertake a formal assessment for coronary artery disease [17]. Apart from our study that addressed this issue, two other studies demonstrated ambivalent results: one study demonstrating higher prevalence of coronary artery disease assessed by abnormal MPIs in patients with AF compared to patients without AF [18], and the other finding no difference between the two groups [17]. An assessment of 150 patients with AF via coronary angiography revealed an increased prevalence of coronary artery disease compared to the prevalence in 148 control patients without AF, suggestive that AF is associated with coronary artery disease [29]. Nevertheless, in another study, despite the presence of a higher prevalence of coronary artery disease in patients with AF, there was no increase in the burden of myocardial ischemia compared to that in non-AF individuals [32]. In addition to the association of AF with the presence of chronic stable CAD, several observational studies have shown an incremental effect on mortality and other major cardiovascular events in patients with acute coronary syndrome (STEMI or NSTEMI) and a history or new onset of AF [33,34,35,36,37].

A systematic review and meta-analysis showed that AF patients had a 1.54-fold increased risk of myocardial infarction induced by CAD [38]. Furthermore, patients with coexistent AF and CAD had a more severe CAD and higher SYNTAX scores compared to those without AF [24]. A recent study also reported that CAD is an independent risk factor for AF after removing all bias [39]. The morbidity and mortality was found to be significantly higher when CAD is associated with AF, and so was the risk of developing heart failure, ventricular arrythmias, and major adverse cardiovascular events (MACEs); however this relationship may be influenced by several confounding risk factors such as diabetes mellitus, hypertension, age, and obesity [30]. Recent evidence also suggests that anthropometric data, including BMI, chest wall conformation, etc., may be related to both the presence of CAD and the presence of AF [40,41,42,43]; on the other hand, BMI has been proven to be an independent predictor of reversible perfusion defects in MPI [44]. Anthropometric parameters may thus mediate the association currently observed between AF and MPI findings.

Whether gating-related artifacts could cause a significantly modified evaluation of perfusion in atrial fibrillation patients is still uncertain; a study with consecutive AF patients who underwent myocardial perfusion SPECT for clinical indications, and had collected both gated and nongated datasets during the same acquisition, reported that AF may have a clinically relevant impact on summed gated perfusion images, compared with images simultaneously obtained without gating in the same patients [45].

In our study, we utilized MPI-SPECT to evaluate the existence of myocardial ischemia in patients with or without AF. All our participants underwent a vasodilator stress test instead of an exercise stress-test, since the presence of AF may reduce the reliability of the MPI after an exercise stress-test used to detect significant CAD [46]. According to our results, a statistically significant difference was found with regard to the existence of myocardial ischemia in patients with AF. A possible explanation for these data could be the relationship between coronary microvascular dysfunction and atrial fibrillation. Ozcan et al. showed that coronary microvascular dysfunction, defined as impaired coronary flow reserve without obstructive coronary disease, is highly prevalent in patients with atrial fibrillation [47]. Not only atrial fibrosis, but also ventricular fibrosis may be correlated with atrial fibrillation, and left ventricular fibrosis may also contribute to ischemic MPI-SPECT results in patients with atrial fibrillation [48]. Shantsila et al. showed that patients with atrial fibrillation had more severe ventricular fibrosis than controls without atrial fibrillation [48]. Moreover, myocardial aging is characterized by left ventricular fibrosis [49], and the older the patient, the more likely it is that they will exhibit signs of atrial fibrillation [50]. Finally, proteins related to inflammation, extracellular remodeling, oxidative stress, and coagulation are associated with the presence of atrial fibrillation and may also indicate that a more complex mechanism plays a role in myocardial ischemia in patients with this arrhythmia [51].

Another study examined the risk of cardiac death in patients with AF utilizing MPI-SPECT, as was the case in our study [18]. Their results indicated that in AF patients, the risk of cardiac death was increased in patients with normal and further increased in those with abnormal MPI-SPECT, reaching 6.4% per year in contrast to 3.7% per year in non-AF patients [18]. They concluded that AF independently increased the risk of deleterious cardiac events in patients undergoing MPI-SPECT, even with slightly abnormal MPI-SPECT. In contrast with our results, the authors did not find any difference in MPI in patients with AF and control group in an article of Gimelli F and Smit MD [19].

Persistent AF has been associated with reduced global hyperemic myocardial blood flow, as measured in MPI positron emission tomography studies, and this could affect the assessment of myocardial perfusion [52,53]. This effect may be reversed with restoration of sinus rhythm following catheter ablation of AF [54], although not consistently [55,56]. Furthermore, baseline and stress myocardial blood flow assessed by cardiac magnetic resonance modality have been shown to be significantly decreased, even in patients with AF but no significant epicardial CAD, and this has been attributed to the severity of underlying left ventricular and left atrial disease (even after successful AF ablation) [57]. In another study comparing the results of an MPI stress test with coronary angiography results, it was shown that the accuracy of MPI stress was mildly reduced in AF vs. non-AF patients, and this was exaggerated in cases where the stress protocol included exercise, but not in the case of a vasodilator test. This difference was attributed to the decreased exercise duration in AF patients [46].

### Limitations

This was a single-center retrospective study, suggesting potential epidemiological associations and not pathogenetic association. The small number of patients was another limitation, and studies involving a larger number of individuals are needed to confirm the conclusions of our study. We cannot exclude the confounding influence of various variables (included or not in statistical analysis), since the two study groups were not matched for them. No echocardiographic data, heart rate data, metabolic parameters, or medication history were consistently recorded during the index test (not used in everyday clinical practice) due to the retrospective nature of the study. For the same reason, only a few variables of the patients’ history were available without further access to the medical records of the whole population. The presence of persistent/permanent AF was based on the ECG during the test and there was no reported history of paroxysmal AF; thus, its association with MPI-SPECT outcomes was not assessed.

## 5. Conclusions

The relationship between AF and CAD is complex, and the two are intricately related at the pathophysiological level. In the current study, an association between AF and extensive perfusion defects in MPI-SPECT studies was found in patients with clinically suspected CAD independently of common cardiovascular risk factors. Patients with atrial fibrillation in our cohort had more atherosclerotic risk factors, which may be related to a higher probability of subclinical epicardial coronary artery disease as well as higher incidence of microvascular dysfunction, which could explain the association with MPI-SPECT results. However, a direct pathophysiological association could not be determined, and one should probably consider AF as a marker of the presence and extent of perfusion defects in MPI-SPECT, while future prospective studies with well-characterized populations are needed to validate our findings and establish a potential causal relationship.

## Figures and Tables

**Table 1:** Descriptive characteristics in patients with vs. without history of AF.

	Without AF N = 169	With AF N = 90	*p* Value
Age, years	61 ± 11	70 ± 9	<0.001
Male gender, n (%)	33 (20)	44 (49)	<0.001
Weight, kg	77 ± 13	78 ± 13	0.447
Smoking, n (%)	41 (24)	32 (36)	0.054
Diabetes, n (%)	39 (23)	28 (31)	0.160
Hypertension, n (%)	119 (70%)	69 (77)	0.283
Dyslipidemia, n (%)	95 (56)	64 (71)	0.019
SSS	2 (1, 4)	5 (3, 9)	<0.001
SRS	0 (0, 2)	3 (1, 6)	<0.001
SDS	1 (0, 2)	3 (0, 4)	0.003
SSS > 3	53 (32)	60 (71)	<0.001
SDS > 1	72 (43)	57 (67)	<0.001

Data are presented as mean ± standard deviation, median (interquartile range) or number (percentage). Abbreviations: AF—atrial fibrillation; SDS—summed difference score; SRS—summed rest score; SSS—summed stress score.

## Data Availability

The data that support the findings of this study are available from the corresponding author upon reasonable request.
